# Case Report: Rare diffuse giant cell tumor of the tendon sheath in the cervical facet joint

**DOI:** 10.3389/fonc.2025.1555098

**Published:** 2025-07-21

**Authors:** Yongliang Jiang, Chongxi Xu, Junpeng Ma, Tianyou Luo

**Affiliations:** ^1^ Department of Neurosurgery, West China Hospital, Sichuan University, Chengdu, Sichuan, China; ^2^ Department of Pathology, West China Hospital, Sichuan University, Chengdu, Sichuan, China

**Keywords:** giant cell tumor of the tendon sheath, cervical spine, vascular and nerve compression, microneurosurgery, recurrence

## Abstract

Giant Cell Tumor of the Tendon Sheath (GCTTS) is a rare neoplasm that can exhibit local aggressiveness but is generally benign. GCTTS is commonly classified into two subtypes: localized and diffuse, and rarely affects the axial skeleton. Diagnosing GCTTS in the spine is challenging, and it is associated with localized pain and neurological symptoms. Surgical resection is now the preferred treatment, though recurrence rates remain high. This report describes an exceptionally rare case of rapidly progressive and extensively invasive diffuse GCTTS affecting the lateral joints, surrounding vasculature, and nerves of the cervical spine. We also review the clinical presentation, imaging characteristics, and therapeutic outcomes of spinal GCTTS to enhance understanding and awareness of this rare pathology.

## Introduction

Giant Cell Tumor of the Tendon Sheath (GCTTS) is a benign neoplasm that originates from synovial cells within bursae, tendon sheaths, and joints. It is typically slow-growing, and patients often seek medical attention two or three years after the onset of the disease. GCTTS predominantly affects tendons in the hands and feet, with the ankle, knee, hip, elbow, and shoulder joints being the most commonly involved (1–3). The occurrence of GCTTS in the spine, however, remains exceedingly rare. Histologically, GCTTS is characterized by synovial cell proliferation, macrophage infiltration, multinucleated osteoclast-like giant cells, and hemosiderin deposition (4, 5). Based on its location and the extent of encapsulation, GCTTS is categorized into two distinct types: localized and diffuse (6, 7). Localized GCTTS typically affects the tendon sheaths of the hands and feet, while diffuse GCTTS demonstrates a more aggressive, infiltrative behavior, often involving the synovium of large joints, such as the knee, hip, ankle, and elbow. It is worth noting that Surgery is the main treatment for GCTTS, but local failure is common, with a local recurrence rate of up to 50% (8–11). In addition, a very small number of cases may progress to malignant lesions, either primary or secondary to long-term recurrent lesions, whose imaging findings overlap with benign GCTTS, and the diagnosis depends on pathological evaluation. The findings are usually polymorphic, with many mitotic signs, including atypical mitosis and extensive necrosis (12–17). Therefore, radical resection and long-term follow-up for such lesions are essential.

Due to the rarity and nonspecific presentation of spinal GCTTS, there have been only sporadic case reports and small case series in the literature over the past few decades (18, 19). This limited number of cases hampers our understanding of its natural history, medical management, and clinical prognosis, making preoperative diagnosis particularly challenging. In this report, we present an exceptionally rare case of diffuse GCTTS in the upper cervical spine, which has extensively eroded the C3–4 lateral joints and compressed the surrounding nerve roots, dura, and vertebral artery. This resulted in persistent neck pain radiating to both shoulders. Following histopathologic confirmation, we review the imaging characteristics, histological features, treatment strategies, and provide a comprehensive review of the existing literature on this uncommon condition. In this study, we optimized the surgical strategy by integrating the imaging and pathological features to provide reference for the management of GCTTS in high-risk areas, and emphasized the key role of total tumor resection in reducing the risk of recurrence and malignant transformation.

## Patient

A 31-year-old right-handed male presented with a six-month history of neck pain, predominantly on the right side, radiating to both shoulders. The patient had no significant medical or surgical history. A needle biopsy of the cervical lesion performed at an external institution revealed focal fibroblastic necrosis, fibrous tissue hyperplasia, and hemosiderin deposition, lacking specific diagnostic features and insufficient to establish a definitive diagnosis, which prompted referral to our institution for further evaluation. The full physical examination and cervical assessment revealed no significant abnormalities. However, palpation of the right cervical spine from C3 to C4 demonstrated marked tenderness. Sensory examination, as well as reflexes in the upper limbs, lower limbs, and abdomen, were all normal. Muscle strength was 5/5, and muscle tone in both upper and lower limbs was within normal limits.

## Imaging findings

**Figure 1:**
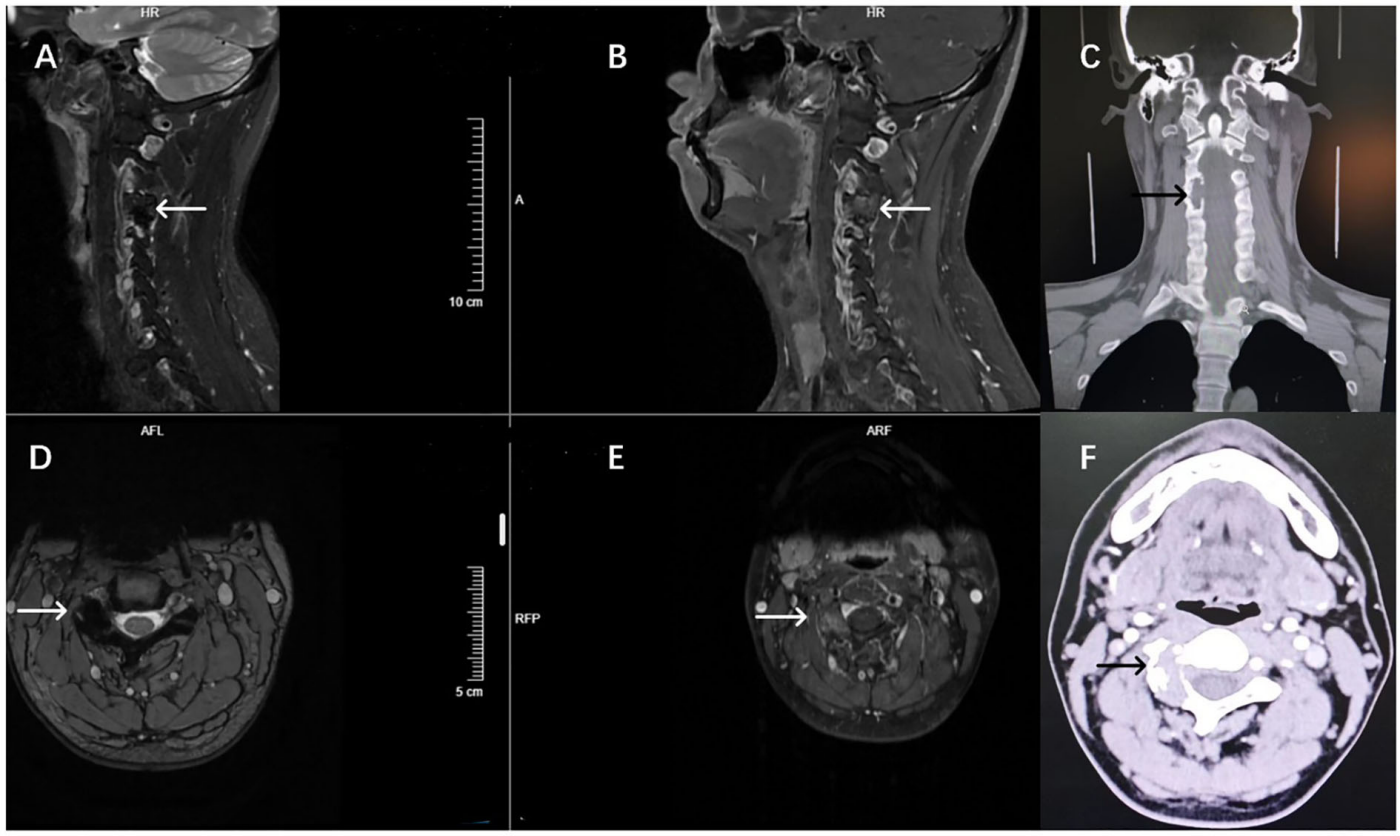
Preoperative imaging findings of the patient. Preoperative MRI T2-weighted imaging showed overall low signal changes **(A, D)**, the C3-4 lateral articular surface disappeared and the overall expansion. T1 enhancement showed uneven enhancement of sagittal and axial plane lesions, complete destruction of the lateral facet joints of the right C3-4, and compression of vertebral artery and neuropore structures **(B, E)**. CT imaging showed mainly dilated bone destruction **(C, F)**.

Three-dimensional CT reconstruction revealed expansile osteolytic destruction of the right C3–4 facet joint, with no significant calcification, measuring approximately 2.2 cm × 1.9 cm × 2.7 cm. MRI showed the lesion with overall low T2 signal intensities, with heterogeneous internal signal. Expansile osteolytic destruction of the right C3–4 facet joint was associated with adjacent bone sclerosis, and part of the lesion extended anteriorly, compressing the right vertebral artery and protruding medially into the C3–4 neural foramen. Post-contrast MRI demonstrated heterogeneous moderate-to-low intensity enhancement, distributed within non-enhancing low-signal areas; the posterior lesion had clear demarcation from adjacent muscle tissue, while the anterior lesion showed ill-defined borders with surrounding tissues (**Figure 1**).

## Operation

**Figure 2:**
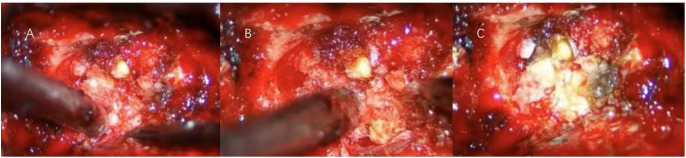
**(A)** During the initial exposure, the boundary between the tumor and the surrounding tissue was blurred, and the surrounding tissue was brittle. **(B)** After sectioning and penetrating into the core of the tumor, the interface between the tumor and the surrounding tissue was gradually created by internal decompression. **(C)** The decompressive interface between the tumor and the surrounding tissue was used to achieve total tumor resection while carefully protecting the nerve and vascular structures.

We proceeded with the planned surgical intervention using standard neurosurgical techniques to excise the space-occupying lesion in the intervertebral foramen. After inducing general anesthesia, the patient was placed in a prone position, and a 15 cm midline posterior incision was made over the C1-C6 vertebrae. The skin, subcutaneous tissue, and muscle were dissected in layers to fully expose the lamina of C2-C5. The compromised right C3-C4 lamina, along with the completely destroyed C3-C4 facet joint, was excised to adequately expose the lesion. The tumor appeared lobulated with a fish-like texture, infiltrating the surrounding tissue and significantly compressing the adjacent nerve roots, right vertebral artery, and dura mater (**Figure 2**). Careful dissection was performed to separate the intradural and extradural tumor masses from the adhesions between the C4 nerve root and the vertebral artery. The tumor was completely resected, ensuring full decompression of the vertebral artery and C4 nerve root. Meticulous hemostasis was achieved in the paravertebral region and lamina, and an autograft and allograft were used to fill the resulting bone defect. Titanium rods and screws were inserted in alignment with the right C2-C5 vertebral pedicles and the left C2 and C5 vertebral pedicles.

## Pathological results

**Figure 3:**
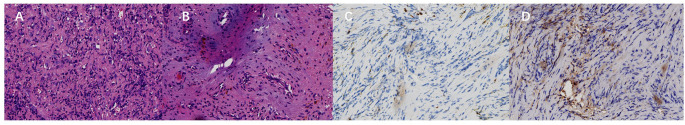
**(A)** Most areas of the tumor show proliferative spindle-shaped and histiocyte-like cells within a fibrous stroma under microscopic examination (hematoxylin-eosin staining, ×400). **(B)** Focal areas demonstrate hemosiderin deposition, ossification, and fibrosis (hematoxylin-eosin staining, ×400). **(C, D)** The lesion is positive for Ki‑67 (5%<, ×400) and CD163 (Envision, ×400). Based on the clinical findings, diffuse Giant cell tumor of tendon sheath was considered.

Pathological examination revealed that most areas of the tumor consisted of proliferative spindle-shaped and histiocyte-like cells within a fibrous stroma. Focal regions exhibited hemosiderin deposition, ossification, and fibrosis. Immunohistochemical staining showed positivity for Ki−67 and CD163 (**Figure 3**). Based on the histopathological and clinical findings, a diagnosis of diffuse giant cell tumor of the tendon sheath was considered.

## Postoperative course

**Figure 4:**
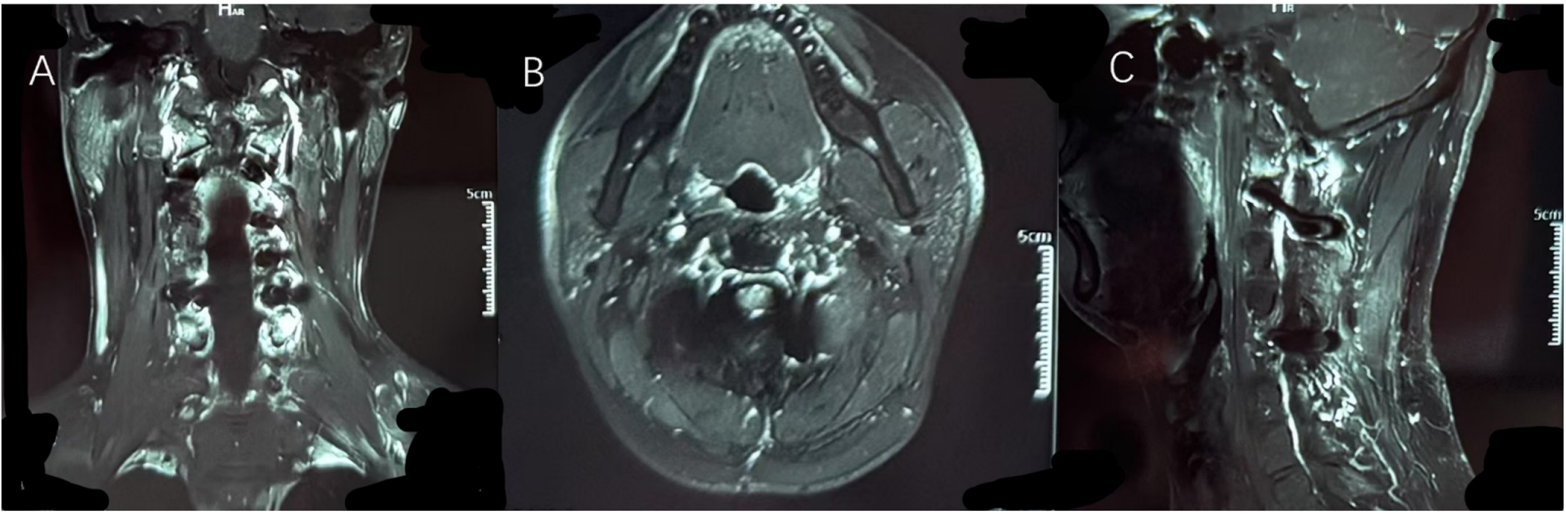
It can be seen from **(A)** coronal position, **(B)** axial position and **(C)** sagittal position that the original tumor area has been completely replaced by autologous bone and artificial bone. Imaging examinations showed no abnormal signals at the edge of the lesion and in the surrounding tissues, and no signs of recurrence of the tumor were observed.

On the second postoperative day, the patient experienced significant relief from neck pain and radiating shoulder pain and was able to ambulate independently. Muscle strength in both upper limbs was rated at 5/5. Routine postoperative CT scans indicated that the screws were securely fixed, with no significant bleeding or further bone destruction at the lesion site. After five days of treatment and observation, the patient was discharged in good health, without the need for further radiotherapy. At the three-month follow-up, neck pain and radiating shoulder pain had resolved, and sensory and motor functions in both upper limbs were intact. The three-month follow-up imaging results indicated normal signals in the surgical cavity and surrounding tissues, with no evidence of recurrence (**Figure 4**). The titanium rods and screws were securely positioned, and spinal stability was deemed satisfactory. We plan to continue regular follow-up for the patient at six and twelve months.

## Discussion

GCTTS is a rare benign soft tissue tumor that rarely affects the axial skeleton, with few reported cases involving the spine. Most clinicians are unfamiliar with its characteristics. The first case of spinal GCTTS was proposed by Kleinman et al. in 1980, with only a limited number of cases reported since. Patients affected by spinal GCTTS span a wide age range, with no clear age predilection (20). Spinal GCTTS often leads to posterior bone destruction, and larger tumors may invade the vertebral body. Clinical presentations vary widely, including asymptomatic cases, pain, and limb weakness, usually correlating with the extent of bone destruction and nerve root compression. The incidence of GCTTS in the cervical spine is particularly high, indicating a greater risk compared to other spinal regions (5, 21). Involvement of the cervical spine poses unique diagnostic and therapeutic challenges due to its proximity to critical neurovascular structures. In this case, the diffuse growth pattern of GCTTS is more destructive and has a higher recurrence rate than the localized growth pattern, affecting paravertebral and epidural tissues and resulting in more complex clinical manifestations (21).

Diffuse giant cell tumor of the tendon sheath (D-GCTTS) is an infiltrative proliferative disorder characterized by synovial or tenosynovial involvement with extra-articular extension, often causing osseous erosion and chronic joint hemorrhage. Typically presenting with insidious progression [2–3 years prior to diagnosis (4, 22–24)], D-GCTTS manifests initially as intermittent localized pain and swelling (4, 23–26), progressing to structural destruction, restricted mobility, and neurological deficits (e.g., radiculopathy/myelopathy) in advanced stages (27, 28). In contrast, this rare upper cervical D-GCTTS case in a 31-year-old healthy male deviated markedly: (1) symptoms were limited to mild, persistent cervical radicular pain without classic neurological dysfunction; (2) rapid symptom progression (6 months) suggested neuroirritative amplification due to spinal anatomical constraints; (3) clinical-imaging dissociation—extensive C3–4 lateral mass destruction on MRI lacked corresponding neurological deficits. Mechanistically, the confined lateral cervical joint space may limit tumor-induced direct neural compression, while heightened pain tolerance in younger patients could mask inflammatory diurnal patterns. This underscores the diagnostic challenge of atypical spinal D-GCTTS, necessitating heightened suspicion for occult neoplasms in patients with unexplained persistent cervical/shoulder pain, even in the absence of trauma, inflammatory markers, or neurological deficits (“triple-negative” profile). Early contrast-enhanced MRI is critical to mitigate diagnostic delay in such cases.

Imaging plays a crucial role in the diagnosis and differentiation of GCTTS from other spinal tumors, with MRI and CT particularly useful for analyzing tumor composition and providing detailed structural information (8). The MRI findings of spinal GCTTS are relatively characteristic but not specific, typically including homogeneous iso- or low signal on T1 and T2-weighted images, reflecting the high hemosiderin content and collagen proliferation within the tumor (29, 30). T2 signal intensity may vary depending on the presence of hemosiderin, fluid, lipids, fibrous tissue, and bleeding (31). On CT, GCTTS typically presents as well-defined lytic lesions with associated bone destruction and without calcification. This characteristic aids in distinguishing it from other spinal tumors, such as metastatic lesions and giant cell tumors of bone (GCTB). When differentiating GCTTS from other spinal lesions, consideration is primarily given to metastatic spinal tumors and GCTs. Although metastatic spinal tumors predominantly affect the posterior elements of the vertebrae, they typically exhibit lytic destruction with ill-defined borders against surrounding tissues, without forming distinct bone separations (32, 33). Due to the presence of multinucleated giant cells, mononuclear cells, and hemosiderin deposits, GCTB is radiographically very similar to GCTTS. However, the characteristic CT appearance of GCTB includes eccentric, expansile lytic lesions with a “soap bubble” appearance, marginal sclerosis, and ossification. Unlike GCTB, which primarily involves tendon sheaths, GCTB most commonly affects the vertebral bodies, particularly in the sacral region, rather than the posterior elements of the vertebrae. These distinctions are critical for guiding treatment and surgical approaches.

Despite its rarity, existing case data and literature reviews indicate that surgical excision remains the gold standard for treating spinal GCTTS. Achieving complete excision of the lesion while clearly delineating its margins is crucial for reducing the risk of recurrence. In previous studies, the recurrence rate after total resection is significantly lower, whereas subtotal resection is associated with a markedly increased risk of tumor regrowth (29, 34). Our compiled data supports (**Supplementary Table 1**). this conclusion: among 70 cases of spinal GCTTS, 9 recurrences occurred after total excision, while 3 out of 5 cases after partial excision recurred, with a chi-square test showing P < 0.05. The extent of surgical resection has been established as the most important prognostic factor for patients with spinal GCTTS. Due to the complex anatomy of the cervical intervertebral foramen, meticulous dissection to protect neurovascular structures while achieving clear separation of the lesion is particularly challenging. In this case, a lesion centered on the cervical lateral joint invaded the intervertebral foramen, prompting us to recommend an extended surgical approach. Utilizing a microscope to carefully identify tumor boundaries allows for maximal resection of the lesion while protecting neural structures. When faced with indistinct margins between the lesion and surrounding tissues, initial decompression within the lesion and segmental excision are vital for clarifying the obscure boundaries with adjacent vascular and neural structures. This technique aims to achieve complete tumor removal while safeguarding crucial neurovascular structures, thereby minimizing recurrence rates. Local recurrence of benign GCTTS is influenced by factors such as the diffuse growth pattern of the tumor, epidural involvement, and soft tissue extension (18), all of which are closely related to local recurrence. In light of this case and literature review, although primary tumors are generally easy to identify and excise, small nodules in the joint space or within the bone may be invisible on imaging and could be overlooked during surgery. If not completely resected, these microscopic satellite lesions may become sources of recurrence (20). This case underwent a biopsy prior to referral to our hospital, a practice we consider inappropriate. While biopsy is an important tool for diagnosing tenosynovial giant cell tumors, its diagnostic application is significantly limited by the complex anatomy of the cervical intervertebral foramen. First, biopsying a tumor that invades the intervertebral structures carries a high risk, as it may damage the surrounding neural roots and vessels. Second, the dense and complex neuroanatomy of this region, combined with the intricate tissue structures within the tumor, limits the ability to obtain comprehensive and in-depth samples, significantly reducing the representativeness and accuracy of the biopsy specimens. Furthermore, small tumor lesions distant from the main tumor can be potential sources of recurrence, and tumor cell dissemination caused by biopsy may increase the risk of recurrence. Considering these limitations, we advise caution in performing biopsies on atypical and rare lesions in primary healthcare institutions, and such patients should be promptly referred to hospitals with comprehensive neuros.

The post-resection reconstruction strategy for the C3–4 bilateral lateral mass defect secondary to GCTTS excision prioritized biomechanical stability while preserving cervical mobility. Lateral mass screw-rod fixation was selected over plate constructs due to its (1) multiplanar adaptability, allowing three-dimensional contouring to match complex cervical anatomy; (2) superior stability with minimal hardware failure risk (35–38); and (3) dynamic adjustability for compression, distraction, and realignment. The osseous void was reconstructed using autologous iliac crest bone graft (primary) supplemented with synthetic bone substitute, leveraging the well-documented osteogenic superiority of autografts (39–42) to expedite fusion and facilitate early collar removal—critical for preventing postoperative stiffness in this young patient. Bilateral C2-C5 lateral mass screws connected via rods were supplemented with left C3-C4 screws in intact lateral masses, creating bilateral mechanical compensation and neutralizing rotational torque through a tension band mechanism (43, 44), thereby enhancing graft stability while mitigating segmental rigidity. This hierarchical reconstruction paradigm—combining rigid screw-rod fixation, bioactive autograft core, and mechanically redundant design—ensures early stability for rapid collar discontinuation while preserving physiological cervical mobility. It establishes a reproducible, function-oriented framework for spinal tumor reconstruction in mobile segments, aligning with enhanced recovery after surgery (ERAS) principles to optimize postoperative rehabilitation quality.

## Conclusion

Our case reports a diffuse tenosynovial giant cell tumor centered on the C3–4 cervical lateral joint, which compresses the intervertebral foraminal nerve roots, anterior vertebral artery, and dura mater. This emphasizes the importance of paying close attention to lesions with indeterminate nature occurring in rare locations, particularly those with unclear relationships to vascular and neural structures. Caution should be exercised in the use of biopsy, a routine diagnostic tool, to avoid potential nerve and vascular injury, as well as the possible dissemination of tumor cells and recurrence due to biopsy procedures. This case also illustrates that a meticulously designed posterior neurosurgical approach can achieve complete excision of the lesion while preserving neural and vascular structures and restoring spinal stability. Given the high recurrence rate associated with diffuse tenosynovial giant cell tumors, long-term close follow-up of patients is warranted, as all these measures contribute to improved long-term health outcomes.

## Data Availability

The raw data supporting the conclusions of this article will be made available by the authors, without undue reservation.
