# Supplementary material for: Case Report: Rare diffuse giant cell tumor of the tendon sheath in the cervical facet joint
Source: Front Oncol. 2025 Jul 21;15:1555098. doi: 10.3389/fonc.2025.1555098 (PMC12318941; doi:10.3389/fonc.2025.1555098)
Supplement: Supplementary file 1 [file DataSheet1.docx]

| **Supplemental table 1: Summary of all well-documented spinal tenosynovial giant cell tumor (TGCT)** | | | | | | | | | |
| --- | --- | --- | --- | --- | --- | --- | --- | --- | --- |
|  | **Sex** | **Age** | **Symptom** | **Loc** | **Fac** | **Epi** | **Treatment** | **F/U** | **R** |
| Pulitzer et al., 1984 [1] | F | 35 | Asymptomatic | C1-4 | ND | ND | GTR | 132m | N |
| Karnezis et al., 1990 [2] | F | 37 | Pain | C6-7 | ND | ND | GTR | 79m | N |
| Khoury et al., 1991 [3] | M | 84 | Myelopathy | C4-5 | Y | ND | GTR | 12m | N |
| Mahmood et al., 1992 [4] | F | 42 | Asymptomatic | C6-7 | Y | ND | ND | ND | ND |
|  | F | 37 | ND | C5 | Y | N | GTR | 108m | N |
|  | F | 37 | ND | C7 | Y | Y | GTR | 66m | N |
|  | M | 38 | ND | C4-5 | Y | Y | GTR | 55m | Y |
| Giannini et al., 1996 [5] | M | 43 | ND | C5 | Y | Y | GTR | 36m | N |
|  | M | 26 | ND | C6 | Y | Y | GTR | 11m | N |
|  | M | 44 | ND | C5-6 | N | Y | GTR | 6m | N |
| Parmar et al., 2004 [6] | M | 43 | Back and neck pain | C5-6 | ND | ND | GTR | 6m | N |
|  | M | 37 | Pain and neurological defect | C3-4 | Y | ND | ND | ND | ND |
|  | M | 31 | Pain | C5-6 | Y | ND | ND | ND | ND |
|  | F | 32 | Pain | C3-4 | Y | ND | ND | ND | ND |
| Motamedi et al., 2005 [7] | M | 23 | Pain and neurological defect | C2-3 | Y | ND | ND | ND | ND |
|  | F | 44 | Pain | C4-5 | Y | ND | ND | ND | ND |
|  | F | 39 | Pain and neurological defect | C5-6 | Y | ND | ND | ND | ND |
|  | ND | ND | ND | C5-6 | Y | ND | ND | ND | ND |
|  | M | 25 | Asymptomatic | C4-5 | Y | ND | ND | ND | ND |
| Finn et al., 2007 [8] | F | 82 | Neck pain, paresthesia ,quadriparesis | C2 | N | ND | Biopsy, stabilization | 24m | N |
| Blankenbaker et al., 2008 [9] | M | 43 | ND | C1 | N | ND | GTR | ND | ND |
| Okutan et al., 2012 [10] | M | 65 | Neck pain, weakness, | C7 | ND | ND | GTR | 6m | N |
| Lavrador et al., 2014 [11] | M | 64 | Asymptomatic | C1-2 | Y | ND | Biopsy | 18m | Residual |
|  | F | 23 | Neck pain | C1-2 | ND | ND | GTR | 132m | N |
| Wang et al., 2014 [12] | F | 33 | Neck pain and hand numbness | C5-7 | ND | ND | GTR | 72m | N |
|  | F | 44 | Neck pain | C1-2 | ND | ND | GTR/En-bloc | 12m | Y |
| Lang et al., 2015 [13] | F | 32 | Neck pain and hand numbness | C1-2 | Y | ND | ND | N | ND |
|  | M | 28 | Neck pain, paresthesias | C4-5 | Y | ND | ND | ND | ND |
|  | F | 33 | Neck discomfort, paresthesias | C5-7 | Y | ND | ND | ND | ND |
|  | F | 22 | Neck masses | C1-2 | Y | ND | ND | ND | ND |
|  | M | 30 | Neck pain | C4 | Y | ND | ND | ND | ND |
| Koontz et al., 2016 [14] | F | 49 | Neck stiffness and spasm, jaw pain | C1-2 | ND | ND | CXT | ND | Residual |
| Kinkor et al., 2016 [15] | F | 59 | Neck pain | C5-6 | ND | ND | ND | ND | ND |
| Furuhata et al., 2019 [16] | F | 32 | Neck pain | C1-2 | ND | ND | GTR | 36m | N |
| Shiliang Cao al., 2023[17] | F | 22 | Mass | C1-2 | ND | ND | GTR/PR | 228m | N |
|  | M | 30 | Pain | C4-5 | ND | ND | GTR/PR | 180m | N |
|  | F | 37 | Pain and neurological defect | C5-7 | ND | ND | GTR/PR | 118m | N |
|  | F | 44 | Pain and neurological defect | C1-3 | ND | ND | STR | 102m | Y |
|  | M | 28 | Pain and neurological defect | C4-6 | ND | ND | STR | 27m | Y |
|  | F | 32 | Pain | C1-2 | ND | ND | STR | 72m | Y |
|  | M | 29 | Pain | C1-2 | ND | ND | GTR | 84m | N |
|  | F | 45 | Pain and neurological defect | C1-2 | ND | ND | GTR | 180m | N |
|  | F | 47 | Pain and neurological defect | C4-6 | ND | ND | GTR | 62m | N |
|  | F | 38 | Asymptomatic | C1 | ND | ND | GTR | 36m | N |
|  | M | 49 | Neurological defect | C1-2 | ND | ND | GTR | 48m | N |
|  | F | 36 | Pain and neurological defect | C3-5 | ND | ND | GTR | 28m | N |
|  | F | 17 | Pain and neurological defect | C6-7 | ND | ND | GTR | 12m | N |
| Piaoe Zeng et al., 2021 [18] | F | 32 | Back pain with left upper numbness for 2 years | C5-7 | Y | N | GTR | 157m | N |
|  | M | 28 | Pain and numbness | C4-5 | N | N | Cytoreductive surgery and postoperative radiotherapy | 76m | N |
|  | F | 32 | Weakness, Neck pain | C1-2 | Y | ND | Cytoreductive surgery and postoperative | 74m | N |
|  | F | 47 | Pain, right upper limb numbness and weakness | C4-6 | Y | N | radiotherapy, GTR and postoperative radiotherapy | 53m | N |
|  | F | 13 | 2 years Left upper limb numb- Benignness for 1.5 months | C5 | N | ND | No treatment | ND | ND |
|  | M | 49 | Numbness or weakness of limbs | C1-2 | Y | ND | Cytoreductive surgery and postoperative radiotherapy | 36m | N |
|  | F | 38 | Asymptomatic | C1 | N | N | GTR and postoperative radiotherapy | 28m | N |
|  | F | 36 | Back pain with numb- Benignness in both hands for 1 year | C4-5 | Y | ND | GTR | 28m | N |
|  | F | 17 | Neck pain for 3 years, upper limbs numbness for 9 months | C6 | N | N | GTR | 8m | N |
|  | F | 31 | Weakness for 2 weeks，then asymptomatic | C6-7 | Y | Y | No treatment | ND | ND |
| Jing-Hui Zhu al., 2021 [19] | M | 49 | Neck pain and weakness of lower extremities | C4-5 | N | Y | Biopsy and GTR | 12m | N |
| Ning Zhu et al., 2021[20] | F | 48 | Headaches | C6-7 | N | N | Biopsy and GTR | 4m | N |
| Hu Y et al., 2021[21] | M | 16 | Numbness of right upper extremity | C2 | N | N | GTR | 12m | N |
| Yun Ji Kim et al., 2021[22] | F | 22 | Asymptomatic | C1-2 | N | N | GTR | 3m | N |
| Rahul Singh al., 2021 [23] | F | 35 | Headaches and facial pain | C1 | N | N | Biopsy and GTR | 1.5m | N |
| Pechlivanis et al., 2009 [24] | M | 35 | ND | C7-T2 | ND | ND | GTR | 24m | N |
| Kuwabara et al., 1991 [25] | F | 25 | Myelopathy | T7-8 | Y | Y | GTR, CXT | 84m | N |
| Clark et al., 1993 [26] | M | 23 | Myelopathy | T7-8 | ND | Y | GTR | 42m | N |
| Gezen et al., 1996 [27] | M | 19 | Myelopathy | T9-12 | Y | Y | STR | 16m | N |
| Giannini et al., 1996 [5] | F | 21 | ND | T3 | Y | N | GTR | 53m | N |
|  | M | 40 | ND | T11 | ND | ND | GTR | 64m | N |
| Clerc et al., 1999 [28] | M | 60 | Back pain, radiculopathy | T9-12 | ND | Y | CXT | ND | ND |
| Bruecks et al., 2001 [29] | F | 13 | Back pain, loss of ambulation and bladder control | T7-8 | ND | Y | GTR | ND | ND |
| Furlong et al., 2003 [30] | F | 21 | ND | T4-5 | Y | Y | GTR | N | ND |
|  | F | 21 | Pain | T4-5 | Y | ND | ND | ND | ND |
| Motamedi et al., 2005 [7] | F | 7 | Pain and neurological defect | T5-6 | Y | ND | ND | ND | ND |
|  | F | 36 | Neurological defect | T5-6 | Y | ND | ND | ND | ND |
|  | M | 30 | ND | T5-6 | N | ND | ND | ND | ND |
| Hansen et al.,2007 [31] | M | 17 | Myelopathy | T6-7 | Y | Y | GTR | N | ND |
| Baena-Ocampo et al., 2009 [32] | M | 17 | Myelopathy | T8-10 | Y | Y | GTR | 72m | N |
| Celiktas et al., 2013 [33] | M | 22 | Paraparesis, back pain | T6-7 | Y | Y | GTR | 24m | N |
| Roguski et al., 2014 [34] | M | 28 | Pain, radiculopathy | T3 | ND | ND | GTR | 6m | N |
| Lang et al., 2015 [13] | M | 22 | Chest and back pain | T11 | Y | ND | ND | ND | ND |
| Sakamoto et al., 2018 [35] | F | 26 | Nuchal pain | T1 | N | ND | Curetted with resection of cortex | 19m | N |
| Yonezawa et al., 2018 [36] | F | 35 | Papillary thyroid CA, otherwise asymptomatic | T6-7 | Y | ND | Frozen recapping laminoplasty, GTR | 36m | N |
| Shen et al., 2019 [37] | F | 39 | Back pain | T9-10 | ND | ND | ND | ND | ND |
| Zeoli et al., 2020 [38] | F | 30 | Back pain, lower body sensation and coordination loss | T4 | Y | Y | GTR | 18m | N |
| Piaoe Zeng et al., 2021 [18] | M | 32 | Thoracic, back and shoulder pain | T1-2 | N | ND | GTR | ND | ND |
|  | M | 44 | Thoracic and back pain | T3 | Y | N | Radiotherapy alone | 36m | N |
|  | M | 45 | Back pain | T9 | N | N | GTR | 117m | Y |
|  | F | 39 | Asymptomatic | T8 | N | N | No treatment | 30m | N |
| Shiliang Cao al., 2023[17] | M | 22 | Pain and neurological defect | T11 | ND | ND | GTR/En-bloc | 60m | N |
|  | M | 25 | Pain and neurological defect | T7-8 | ND | ND | GTR | 120m | N |
|  | F | 26 | Pain and neurological defect | T2-3 | ND | ND | GTR | 39m | N |
|  | M | 32 | Pain | T1-2 | ND | ND | GTR | 12m | N |
|  | M | 46 | Pain | T9 | ND | ND | GTR/En-bloc | 141m | Y |
| Chengru Song et al., 2023 [39] | F | 28 | Lower limb weakness | T9-10 | N | Y | GTR | ND | ND |
| Campbell et al., 1982 [40] | F | 54 | Pain | L4-5 | ND | ND | GTR | 31m | N |
| Titelbaum et al., 1992 [41] | F | 52 | Radiculopathy | L4-5 | ND | ND | GTR | ND | N |
| Weidner et al., 1986 [42] | F | 34 | Back pain, paresthesia | L2-5 | Y | ND | STR | 12m | N |
| Khoury et al., 1991 [3] | F | 61 | Buttock, hip, and leg pain | L4-5 | Y | ND | GTR | ND | ND |
| Giannini et al., 1996 [5] | M | 42 | ND | L3 | ND | ND | STR | 14m | Residual |
|  | M | 67 | ND | L3 | Y | Y | ND | ND | ND |
| Dimeco et al., 2001 [43] | F | 70 | Radiculopathy | L3-4 | ND | ND | GTR | 12m | N |
| Motamedi et al., 2005 [7] | F | 25 | ND | L4-5 | Y | ND | ND | ND | ND |
|  | F | 29 | ND | L4-5 | Y | ND | ND | ND | ND |
| Oe et al., 2007 [44] | M | 43 | Back pain | L4-5 | Y | N D | GTR | 36 | N |
| Musluman et al., 2008 [45] | F | 59 | Back pain | L4 | Y | ND | GTR | 6m | N |
| Rovner et al., 2008 [46] | F | 37 | Neck and bac pain, paresthesia, | L5 | Y | ND | GTR/En-bloc | 8m | N |
| Yener et al., 2010 [47] | F | 66 | Back pain | L2 | Y | Y | GTR | 2m | N |
| Hsieh et al., 2012 [48] | M | 39 | Low back pain, paresthesia | L2-3 | Y | ND | GTR | 3m | Y |
| Oh et al., 2014 [49] | M | 38 | Bilateral low back pain | L4 | ND | Y | GTR | 24m | N |
| Kimura et al., 2015 [50] | F | 14 | Back pain | L5 | Y | ND | GTR | ND | ND |
| Lang et al., 2015 [13] | M | 54 | Low back pain | L4-5 | ND | ND | ND | ND | ND |
| Weidner et al., 1986 [42] | F | 48 | Back pain | L5-S1 | Y | ND | GTR | 10m | Y |
| Giannini et al., 1996 [5] | F | 29 | ND | L5-S1 | Y | N | GTR | 23m | Y |
| Motamedi et al., 2005 [7] | F | 14 | Pain | L5-S1 | Y | ND | ND | ND | ND |
| Oda et al., 2007 [51] | F | 53 | Paresthesias, buttock pain | L5-S1 | Y | ND | GTR | 17m | Y |
| Loc = location and spinal level, Fac = facet involvement, Epi = epidural involvement, F/U = follow-up, R = recurrence, M = male, F = female，STR = subtotal resection, GTR = gross total resection, PR= Piecemeal resection, En-bloc=en-bloc resection, CXT = chemotherapy, m = months, ND = no data, Y = yes, N = no. | | | | | | | | | |

**Supplementary References：**

**[1] D.R. Pulitzer, R.J. Reed, Localized pigmented villonodular synovitis of the vertebral column, Arch Pat hoi Lab Med. 108 (1984) 228–230.**

**[2] T.A. Karnezis, R.D. McMillan, I. Ciric, Pigmented villonodular synovitis in a vertebra. A case report, J. Bone Joint Surg. Am. 72 (6) (1990) 927–930.**

**[3] G.M. Khoury, P.M. Shimkin, G.M. Kleinman, P.P. Mastroianni, D.E. Nijensohn, Computed tomography and magnetic resonance imaging findings of pigmented villonodular synovitis of the spine, Spine (Phila Pa 1976). 16 (10) (1991) 1236–1237, https://doi.org/10.1097/00007632-199110000-00018.**

**[4] A. Mahmood, D.V. Caccamo, J.K. Morgan, Tenosynovial giant-cell tumor of the cervical spine. Case report, J. Neurosurg. 77 (6) (1992) 952–955, https://doi.org/ 10.3171/jns.1992.77.6.0952.**

**[5] Giannini C, Scheithauer BW, Wenger DE, Unni KK. Pigmented villonodular synovitis of the spine: a clinical, radiological, and morphological study of 12 cases. J Neurosurg. 1996 Apr;84(4):592-7. doi: 10.3171/jns.1996.84.4.0592**

**[6] H.A. Parmar, Y.Y. Sitoh, K.K. Tan, J. Teo, S.M. Ibet, F. Hui, MR imaging features of pigmented villonodular synovitis of the cervical spine, AJNR Am. J. Neuroradiol. 25 (1) (2004) 146–149.**

**[7] Motamedi K, Murphey MD, Fetsch JF, Furlong MA, Vinh TN, Laskin WB, Sweet DE. Villonodular synovitis (PVNS) of the spine. Skeletal Radiol. 2005 Apr;34(4):185-95. doi: 10.1007/s00256-004-0880-9**

**[8] M.A. Finn, T.D. McCall, M.H. Schmidt, Pigmented villonodular synovitis associated with pathological fracture of the odontoid and atlantoaxial instability. Case report and review of the literature, J. Neurosurg. Spine 7 (2) (2007) 248–253, https://doi.org/10.3171/SPI-07/08/248.**

**[9] Blankenbaker DG, Tuite MJ, Koplin SA, Salamat MS, Hafez R. Tenosynovial giant cell tumor of the posterior arch of C1. Skeletal Radiol. 2008 Jul;37(7):667-71. doi: 10.1007/s00256-008-0459-y**

**[10] O. Okutan, I. Solaroglu, O. Ozen, B. Saygili, E. Beskonakli, Tenosynovial giant cell tumor in the cervico-thoracic junction, Turk. Neurosurg. 22 (6) (2012) 769–771, https://doi.org/10.5137/1019-5149.JTN.315-07.3.**

**[11] J.P. Lavrador, E. Oliveira, N. Gil, A.F. Francisco, S. Livraghi, C1-C2 pigmented villonodular synovitis and clear cell carcinoma: unexpected presentation of a rare disease and a review of the literature, Eur. Spine J. 24 (Suppl 4) (2015) S465–S471, https://doi.org/10.1007/s00586-014-3396-6.**

**[12] K. Wang, B. Zhu, S. Yang, Z. Liu, M. Yu, X. Liu, Primary diffuse-type tenosynovial giant cell tumor of the spine: a report of 3 cases and systemic review of the literature, Turk. Neurosurg. 24 (5) (2014) 804–813, https://doi.org/10.5137/1019-5149.JTN.9594-13.1.**

**[13] N. Lang, H.S. Yuan, Computed tomography and magnetic resonance manifestations of spinal pigmented villonodular synovitis, J. Comput. Assist. Tomogr. 39 (4) (2015) 601–606, https://doi.org/10.1097/RCT.0000000000000244.**

**[14] N.A. Koontz, E.P. Quigley, B.L. Witt, R.K. Sanders, L.M. Shah, Pigmented villonodular synovitis of the cervical spine: case report and review of the literature, BJR Case Rep. 2 (1) (2015), 20150264, https://doi.org/10.1259/bjrcr.20150264.Published 2015 Oct 12.**

**[15] Z. Kinkor, T. Svoboda, P. Grossman, et al., Difúzníobrovskobunˇeˇcný tumor slachovýchpochevkrˇcníp´ateˇre s destrukcíobratle C6 - kazuistika [Diffuse tenosynovial giant cell tumor of the cervical spine destroying vertebra C6 - a case report], CeskPatol. 52 (4) (2016) 218–221.**

**[16] R. Furuhata, A. Iwanami, O. Tsuji, et al., Tenosynovial giant cell tumor of the cervical spine: a case report, Spinal Cord Ser. Cases 5 (1) (2019) 23, https://doi.org/10.1038/s41394-019-0172-1**

**[17] Cao S, Jiang L, Yang S, Liu Z, Wei F, Liu X. Surgical treatment of spinal tenosynovial giant cell tumor: Experience from a single center and literature review. Front Oncol. 2023 Jan 17;12:1063109. doi: 10.3389/fonc.2022.1063109**

**[18] Zeng P, Zhang A, Song L, Liu J, Yuan H, Zhang W. Giant cell tumour of the tendon sheath of the spine: clinical features and imaging findings. Insights Imaging. 2021 Jul 13;12(1):98. doi: 10.1186/s13244-021-01025-2**

**[19] Zhu JH, Li M, Liang Y, Wu JH. Tenosynovial giant cell tumor involving the cervical spine: A case report. World J Clin Cases. 2021 May 16;9(14):3394-3402. doi: 10.12998/wjcc.v9.i14.3394**

**[20]** **Zhu N, Campbell R, Sadasivan AP. Tenosynovial giant cell tumours of the upper and lower cervical spine: two case reports. Spinal Cord Ser Cases. 2022 Aug 3;8(1):72. doi: 10.1038/s41394-022-00538-2IF**

**[21] Hu Y, Chen M, Richard SA, Huang S. Localized Giant Cell Tumor of the Tendon Sheath of the Upper Cervical Spine: A Case Report. Neurol India. 2022 Mar-Apr;70(2):764-766. doi: 10.4103/0028-3886.344600**

**[22] Kim YJ, Hong JH, Park JH, Cho SJ. Tenosynovial giant cell tumor of the upper cervical spine arising from the posterior atlanto-occipital membrane: a case report. Skeletal Radiol. 2021 Feb;50(2):451-455. doi: 10.1007/s00256-020-03569-8**

**[23] Singh R, Stienen MN, Ganjoo K, Kolahi KS, Cayrol R, Charville GW, Born DE, Zygourakis CC. Tenosynovial giant cell tumor of the suboccipital region - A rare, benign neoplasm in this location. J Clin Neurosci. 2020 Aug;78:413-415. doi: 10.1016/j.jocn.2020.05.022**

**[24] Pechlivanis I, Tannapfel A, Tüttenberg J, Harders A, Schmieder K. Pigmentierte villonoduläre Synovitis der thorakalen Wirbelsäule - operatives Langzeitergebnis: ein Fallbericht [Pigmented villonodular synovitis involving the thoracic spine: a case report]. Z Orthop Unfall. 2009 Mar-Apr;147(2):220-4. German. doi: 10.1055/s-0029-1185526**

**[25] Kuwabara H, Uda H, Nakashima H. Pigmented villonodular synovitis (giant cell tumor of the synovium) occurring in the vertebral column. Report of a case. Acta Pathol Jpn. 1992 Jan;42(1):69-74. doi: 10.1111/j.1440-1827.1992.tb01113.x**

**[26] Clark LJ, McCormick PW, Domenico DR, Savory L. Pigmented villonodular synovitis of the spine. Case report. J Neurosurg. 1993 Sep;79(3):456-9. doi: 10.3171/jns.1993.79.3.0456**

**[27] Gezen F, Akay KM, Aksu AY, Bedük A, Seber N. Spinal pigmented villonodular synovitis: a case report. Spine (Phila Pa 1976). 1996 Mar 1;21(5):642-5. doi: 10.1097/00007632-199603010-00021**

**[28] Clerc D, Berge E, Benichou O, Paule B, Quillard J, Bisson M. An unusual case of pigmented villonodular synovitis of the spine: benign aggressive and/or malignant? Rheumatology (Oxford). 1999 May;38(5):476-7. doi: 10.1093/rheumatology/38.5.476**

**[29] Bruecks AK, Macaulay RJ, Tong KA, Goplen G. November 2000: 13 year old girl with back pain and leg weakness. Brain Pathol. 2001 Apr;11(2):263-4. PMID: 11303802**

**[30] Furlong MA, Motamedi K, Laskin WB, Vinh TN, Murphey M, Sweet DE, Fetsch JF. Synovial-type giant cell tumors of the vertebral column: a clinicopathologic study of 15 cases, with a review of the literature and discussion of the differential diagnosis. Hum Pathol. 2003 Jul;34(7):670-9. doi: 10.1016/s0046-8177(03)00250-8**

**[31] Hansen MA, Harper C, Yiannikas C, McGee-Collett M. A rare presentation of pigmented villonodular synovitis. J Clin Neurosci. 2007 Apr;14(4):386-8. doi: 10.1016/j.jocn.2005.12.013**

**[32] del Carmen Baena-Ocampo L, Rosales Olivares LM, Arriaga NM, Izaguirre A, Pineda C. Pigmented villonodular synovitis of thoracic facet joint presenting as rapidly progressive paraplegia. J Clin Rheumatol. 2009 Dec;15(8):393-5. doi: 10.1097/RHU.0b013e3181c3f894**

**[33] Celiktas M, Asik MO, Gezercan Y, Gulsen M. Pigmented villonodular synovitis of the thoracic vertebra presenting with progressive spastic paraparesis. Case Rep Orthop. 2013;2013:870324. doi: 10.1155/2013/870324**

**[34] Roguski M, Safain MG, Zerris VA, Kryzanski JT, Thomas CB, Magge SN, Riesenburger RI. Pigmented villonodular synovitis of the thoracic spine. J Clin Neurosci. 2014 Oct;21(10):1679-85. doi: 10.1016/j.jocn.2014.03.012**

**[35] Sakamoto A, Matsuyama A, Hisaoka M, Matsuda S. Bone Involvement Mimicking an Aggressive Bone Lesion in a Diffuse-type Tenosynovial Giant Cell Tumor in the Thoracic Vertebral Lamina: A Case Report. J Orthop Case Rep. 2018 May-Jun;8(3):14-17. doi: 10.13107/jocr.2250-0685.1088**

**[36] N. Yonezawa, H. Murakami, S. Kato, H. Hayashi, H. Tsuchiya, Successful treatment of a diffuse type tenosynovial giant cell tumor in the thoracic spine mimicking spinal metastasis by frozen recapping laminoplasty in a patient with thyroid cancer, Eur. Spine J. 27 (Suppl 3) (2018) 526–532, https://doi.org/10.1007/s00586-018-5603-3.**

**[37] G. Shen, H. Ma, L. Pan, M. Su, A. Kuang, Diffuse-type tenosynovial giant cell tumor of the thoracic spine: appearance on FDG PET/CT, Clin. Nucl. Med. 44 (8) (2019)e477–e478,** [**https://doi.org/10.1097/RLU.0000000000002541**](https://doi.org/10.1097/RLU.0000000000002541)

**[38] Zeoli T, Mathkour M, Scullen T, Carr C, Abou-Al-Shaar H, Wang L, Divagaran A, Dindial R, Tubbs RS, Bui CJ, Maulucci CM. Spinal pigmented villonodular synovitis and tenosynovial giant cell tumor: A report of two cases and a comprehensive systematic review. Clin Neurol Neurosurg. 2021 Mar;202:106489. doi: 10.1016/j.clineuro.2021.106489**

**[39] Song C, Xie S, Cheng J. Giant cell tumor of tendon sheath in thoracic spinal canal: A case report. Asian J Surg. 2023 Nov;46(11):5217-5218. doi: 10.1016/j.asjsur.2023.07.016**

**[40] A.J. Campbell, I.P. Wells, Pigmented villonodular synovitis of a lumbar vertebral facet joint, J. Bone Joint Surg. Am. 64 (1) (1982) 145–146.**

**[41] D. Titelbaum, C.H. Rhodes, J.S. Brooks, H.I. Goldberg, Pigmented villonodular synovitis of a lumbar facet joint, Am. J. Neuroradiol. 13 (1992) 164–166.**

**[42] N. Weidner, V.R. Challa, S.M. Bonsib, C.H. Davis Jr., T.J. Carrol Jr., Giant cell tumors of synovium (Pigmented villonodular synovitis) involving the vertebral column, Cancer. 57 (10) (1986) 2030–2036,** [**https://doi.org/10.1002/1097-0142**](https://doi.org/10.1002/1097-0142)

**[43] Dimeco F, Rizzo P, Li KW, Ciceri E, Casali C, Pollo B, Lasio G. Pigment villonodular synovitis of the spine. Case report and review of the literature. J Neurosurg Sci. 2001 Dec;45(4):216-9; discussion 219.**

**[44] K. Oe, K. Sasai, Y. Yoshida, et al., Pigmented villonodular synovitis originating from the lumbar facet joint: a case report, Eur. Spine J. 16 (Suppl 3(Suppl 3))(2007) 301–305, https://doi.org/10.1007/s00586-007-0403-1.**

**[45] A.M. Müslüman, H. Cavus¸o˘glu, A. Yilmaz, T. Dalkiliç, C. Tanik, Y. Aydin, Pigmented villonodular synovitis of a lumbar intervertebral facet joint, Spine J. 9(8) (2009) e6–e9, https://doi.org/10.1016/j.spinee.2008.12.010.**

**[46] J. Rovner, A. Yaghoobian, M. Gott, N. Tindel, Pigmented villonodular synovitis of the zygoapophyseal joint: a case report, Spine (Phila Pa 1976) 33 (18) (2008)E656–E658,**[**https://doi.org/10.1097/BRS.0b013e31817eb85a**](https://doi.org/10.1097/BRS.0b013e31817eb85a)**.**

**[47] Yener U, Konya D, Bozkurt S, Ozgen S. Pigmented villonodular synovitis of the spine: report of a lumbar case. Turk Neurosurg. 2010 Apr;20(2):251-6. doi: 10.5137/1019-5149.JTN.1590-08.3**

**[48] Hsieh YC, Chen WY, Hsieh TY, Chan WP. Pigmented villonodular synovitis of the lumbar spine. J Clin Rheumatol. 2012 Aug;18(5):274-5. doi: 10.1097/RHU.0b013e318264215a**

**[49] Oh SW, Lee MH, Eoh W. Pigmented villonodular synovitis on lumbar spine : a case report and literature review. J Korean Neurosurg Soc. 2014 Sep;56(3):272-7. doi: 10.3340/jkns.2014.56.3.272**

**[50] T. Kimura, T. Nishisho, T. Sakai, et al., Tenosynovial giant cell tumor, diffuse Type/Pigmented villonodular synovitis in a pars defect: a case report, Spine (Phila Pa1976) 40 (12) (2015) E735–E739,** [**https://doi.org/10.1097/BRS.0000000000000923**](https://doi.org/10.1097/BRS.0000000000000923)

**[51] Y. Oda, T. Takahira, R. Yokoyama, M. Tsuneyoshi, Diffuse-type giant cell tumor/pigmented villonodular synovitis arising in the sacrum: malignant form, Pathol.Int. 57 (9) (2007) 627–631,**[**https://doi.org/10.1111/j.1440-1827.2007.02150.x**](https://doi.org/10.1111/j.1440-1827.2007.02150.x)

| **Supplemental table 2: The baseline of all well-documented spinal tenosynovial giant cell tumor (TGCT)** | | | |
| --- | --- | --- | --- |
| Variable | **Cervical** | **Thoracic and cervicothoracic junction** | **Lumbar and lumbosacral junction** |
| Cases | 62 | 32 | 21 |
| Average age | 37.4 | 28.9 | 44.1 |
| Male | 40% | 56.25% | 71% |
| Female | 60% | 43.75% | 29.00% |
| Presentation |  |  |  |
| Sensory | Pain，paresthesia or numbness(77.8%) | Pain，paresthesia or numbness(88.9%) | Pain，paresthesia or numbness(81.5 %) |
| Motory | Weakness, cervical myelopathy, or other neurological defect(27.8%) | Weakness, thoracic myelopathy or other neurological defect(55.56%) | Radiculopathy (37.5 %) |
| Else | Asymptomatic (13%) | Asymptomatic (3%) | Asymptomatic (0) |
| Facet involvement | 69.20% | 70.00% | 94% |
| Dural involvement | 41.20% | 73.30% | 75% |
| GTR | 77% | 80.77% | 87.50% |
| Recurrence | 10.40% | 10% | 30.70% |
